# Complete Genome Sequence of Bacteriophage Finny, Isolated from a Microbacterium foliorum Culture

**DOI:** 10.1128/MRA.01039-19

**Published:** 2019-10-03

**Authors:** Tiffany Lee, Michaela Aguirre, Shey Andrews, Kayla Bahr, Abigail Ballard, Matthew Bristerpostma, Faith Cox, Leah Dowell, David Kiker, Tiffany Lujan, Stacy Luka, Abbigal Ramirez, Rheaven Sandoval, Kenneth Underhill, Haze Murphy, Cecilia Cabrera, Dustin Edwards

**Affiliations:** a Department of Biological Sciences, Tarleton State University, Stephenville, Texas, USA; Queens College

## Abstract

Actinobacteriophage Finny contains a circularly permuted 40,313-bp double-stranded DNA genome with 63 predicted protein-coding genes. Finny was directly isolated from a soil sample collected in New Braunfels, Texas, that was incubated with Microbacterium foliorum SEA B-24224. Finny is closely related to bacteriophages MCubed, Andromedas, ColaCorta, Eleri, and Sansa.

## ANNOUNCEMENT

Characterization of actinobacteriophage genomes provides valuable information on virus evolution and genetic diversity, including conservation of shared genes within a population (1). We report the genome sequence of actinobacteriophage Finny (2), directly isolated from soil inside a chicken coop in New Braunfels, TX (global positioning system [GPS] coordinates 29.782774N, 98.128783W). Soil samples were washed with peptone-yeast extract-calcium (PYCa) liquid medium and filtered through 0.22-μm filters to extract the bacteriophage. The filtered medium was plated using a softagar overlay method on PYCa agar with Microbacterium foliorum strain SEA B-24224 at 29°C for 24 h. Bacteriophage replication formed small and medium lytic plaques with turbid halo rings. Finny was purified by picking a single plaque and placing it in 100 μl phage buffer (10 mM Tris [pH 7.5], 10 mM MgSO_4_, 68 mM NaCl, 1 mM CaCl_2_, 10% glycerol), followed by two consecutive serial dilutions. Negative-staining transmission electron microscopy showed that the isolated bacteriophage had siphoviral morphology, with an approximate tail length of 150 nm and capsid diameter of 65 nm.

The bacteriophage DNA was extracted by a modified zinc chloride precipitation method (3) that included proteinase K (20 mg/ml) treatment to deactivate the nucleases prior to potassium acetate precipitation. Genomic sequencing libraries were prepared using the NEBNext Ultra II kit (New England BioLabs, Ipswich, MA); the libraries were pooled and sequenced with an Illumina MiSeq instrument at the Pittsburgh Bacteriophage Institute (Pittsburgh, PA). Sequencing was performed to approximately 7,918-fold coverage from 2,253,035 total single-end 150-base read length (4). A single bacteriophage contig was assembled from sequence reads using Newbler 2.9, with default settings, and Consed v29.0 (5) was used to perform quality control on assembly for inclusiveness and precision. We determined that the virus contains a double-stranded DNA genome 40,313 base pairs long, with 62.1% GC content. There were no accumulations of read starts or substantial coverage variations, so the genome was determined to be circularly permuted. The beginning of the genome was chosen by comparison to similar bacteriophage genomes.

Whole-genome nucleotide alignment with BLASTn (https://blast.ncbi.nlm.nih.gov/) (6) showed similar nucleotide identity to the bacteriophages summarized in Table 1 (2, 7).

**TABLE 1:** Characteristics of similar bacteriophages with M. foliorum as a host^*a*^

Name	GenBank accession no.	Genome size (bp)	GC content (%)	No. of ORFs^b^	No. of tRNAs	% identity to Finny
Finny	MK894432	40,313	62.1	63	1	
MCubed	MN096378	40,381	62.0	63	0	97.44
Andromedas	MH590606	40,494	62.0	63	1	96.86
Eleri	MG839027	40,366	62.0	63	0	96.82
ColaCorta	MH590604	40,494	62.0	64	1	96.80
Sansa	MH513982	40,306	61.8	62	1	96.41

a Similarity defined as an identity of >95%.

b ORFs, open reading frames.

Along with manual inspection, genome annotation was performed using GLIMMER v3.02 (8) and GeneMark v2.5p (9, 10) for correction and refinement of start sites and revisions utilizing Phamerator (https://phamerator.org/) (11), DNA Master v5.23.2 (http://phagesdb.org/DNAMaster/), and PECAAN (https://discover.kbrinsgd.org). We predicted that Finny contained 63 protein-coding genes. One tRNA gene was identified by tRNAscan-SE v2.0 (12) and positioned at nucleotides 28924 through 28857. Start codon usage was determined to be 80.95% AUG and 19.05% GUG. Putative functions for 25 of the 63 predicted protein-coding genes were assigned using HHpred v3.0beta (13, 14) and NCBI BLASTp (6). The Finny genome is organized with genes 1 through 26 transcribed rightwards and encoding terminase, lysin A, and virion assembly and structural proteins. Genes transcribed leftwards encode RecA-like DNA recombinase, AAA-ATPase, Cas4 family exonuclease, DNA polymerase I, DNA helicase, MazG-like nucleotide pyrophosphohydrolase, thymidylate kinase, glycosyltransferase, and ThyX thymidylate synthase.

### Data availability.

The genome sequence of actinobacteriophage Finny is available at GenBank under accession number MK894432. The raw reads are available in the SRA under accession number SRX6700907.
